# Factors Associated with Housing Damage Caused by an EF4 Tornado in Rural Areas of Funing, China

**DOI:** 10.3390/ijerph192114237

**Published:** 2022-10-31

**Authors:** Peng Qiao, Wei Chen, Jun Zhao, Jingyi Gao, Guofang Zhai

**Affiliations:** 1 School of Geographic and Oceanographic Sciences, Nanjing University, Nanjing 210046, China; 2 School of Geographic and Biologic Information, Nanjing University of Posts and Telecommunications, Nanjing 210023, China; 3 Jiangsu Provincial Architectural Design and Research Institute, Nanjing 210019, China; 4 Graduate School of Engineering, Tohoku University, Sendai 980-8572, Japan

**Keywords:** tornado, housing damage, wind disaster, emergency management, disaster prevention

## Abstract

Rural areas are vulnerable to natural disasters and tend to suffer severe losses. An EF4 tornado occurred in Funing on 23 June 2016, killing 99 people, injuring at least 846 people, and destroying more than 2000 houses. Using a multinomial logistic regression model, this study explored the influencing factors between housing damage and variables of building conditions, tornado intensity, and village environmental factors. The results show that 2-story houses and masonry houses were more likely to be slightly damaged or be in a dangerous state. Furthermore, the building area was positively related to houses in two categories: slight damage (SD) and dangerous and requiring immediate repair (DR), indicating that the larger or taller the house, the more severe the damage. In terms of tornado intensity, houses classified as SD were more likely to be hit by EF4 tornados than by EF3 tornados, and houses were damaged more by EF1 or EF2 tornados. This finding demonstrates that the level of housing damage was not strongly correlated with the tornado intensity. Slightly damaged houses exhibited the highest correlation with environmental factors. The proportion of slightly damaged houses was positively correlated with the water area in the village, unlike the proportion of houses in the DR and unable to be repaired (UR) categories. Moreover, the larger the water area of a village, the less housing damage it suffered. These findings provide new insights into minimizing housing damage in wind disasters to improve disaster prevention planning in rural areas.

## 1. Introduction

Natural disasters, particularly devastating weather-related disasters, cause significant concern worldwide. Approximately 80% of economic losses resulting from natural disasters are caused by extreme winds and associated events [1,2]. The World Meteorological Organization (WMO) reported that one weather, climate, or water-related disaster has occurred on average every day during the past 50 years, taking the lives of 115 people and causing USD 202 million in losses daily [3]. In China, tornados are considered the most violent atmospheric hazard [4]. They are a common weather phenomenon [5], with approximately 108(±44) tornados every year, resulting in heavy losses to personnel, the economy, and the environment [6]. Regarding spatial distribution, tornadoes in China mainly occur in the eastern plains, of which the middle and lower reaches of the Yangtze River are the frequent areas. According to statistical data from the National Climate Center from 1991 to 2020, China has an average of 38 tornadoes every year. Provinces such as Jiangsu, Guangdong, Hubei, and Anhui have a large number of tornadoes, with Jiangsu and Guangdong having the largest number. The annual average number of tornadoes is 4.8 and 4.3 respectively, followed by Hubei and Anhui, both of which have two. From the time distribution, the number of tornadoes from April to August accounted for 92% of the year, with July accounting for the highest proportion of about 30%. Judging from the occurrence time of strong tornadoes of EF2 and above, it also has the most in July [7]. China began researching tornados in the 1980s, much later than in developed countries. Currently, scholars are exploring the climate mechanism of tornados and performing tornado monitoring on the regional to the national scale [8]. Although the frequency of tornado occurrence has decreased consistently since the 1980s, several severe tornados have occurred in China in recent years [9]. Due to the huge losses to the environment and society, significant efforts have been made to deal with these disasters. The losses to the ecosystems, economy, personnel, and buildings caused by wind-related disasters have been studied [10,11,12]. Among all of these, the vulnerability of buildings is a crucial issue in the analysis of wind-induced physical damage [13]. In particular, housing damage is a great concern due to casualties, resettlement, and post-disaster reconstruction [14,15]. However, information on the factors affecting housing damage is limited [16,17,18]. Analyzing the factors influencing housing damage in tornados enables us to learn from disasters and reduce losses by improving disaster prevention measures.

A building damage survey can provide basic information on the disaster resilience of buildings and facilitate the analysis of building damage [19,20]. The building type has been used as a variable for evaluating hurricane-induced building damage [21,22]. Huo et al. [23] investigated buildings damaged by Typhoon Soudelor in Chinese coastal areas, revealing common characteristics of the damaged houses such as inappropriate roof materials, aged structures, and the lack of integrity. Wind-induced housing damage includes exterior and interior damage. For low-rise buildings, the wind speed, exterior housing damage, and building shape have been used as variables for analyzing damage [24]. The building structure is a key variable in damage investigations and vulnerability analysis. Light structures such as wood-frame houses are more likely to be damaged by hurricanes and earthquakes [25]. The wind speed uncertainty is crucial in wind disaster analysis. In a tornado damage survey at Moore, Oklahoma, the wind speeds that damaged the residential structures were significantly lower than the established F-scale wind speeds, suggesting that low wind speeds during a tornado can cause severe building damage [26]. Kwon et al. [27] considered wind speed, building damping ratio, and terrain conditions in non-hurricane and hurricane winds. They found that the load factors were higher when the uncertainty in estimating the hurricane wind speed was accounted for [28]. Morrison et al. [29] investigated tornado-damaged houses in Vaughan, Ontario, and found that roof failure was due to internal pressurization and defects in the connections between the roof and the walls. Eight characteristics of buildings (roof, exterior wall, story number, year built, building area, floor, foundation, and shape) were used as indicators to establish a hurricane loss model. The higher the wind speed, the more building debris was produced, and the greater the external damage [30]. In addition, the building height is also a crucial indicator of building safety during wind-induced events. Numerous studies have analyzed the response of tall buildings to wind events [10,12]. Hu et al. [31] analyzed coastal topographic factors, community factors, buildings, and condition data on hurricane damage to residential structures. The results revealed that 1-story buildings were the most vulnerable to damage, but buildings with two or more stories were most likely to survive in a hurricane event. Pan et al. [32] found that wind-induced internal and external pressures contributed to the net pressure in low-rise buildings with multiple openings. Failures of roofs and walls are common, especially in smaller residential buildings [33].

Studies have shown that residential buildings in rural areas have less resilience and are easily damaged during severe natural hazards. Xie et al. [34] investigated rural buildings damaged by a strong wind, revealing that rural houses were highly vulnerable to strong wind events. Zaini et al. [35] found that rural houses were more vulnerable during windstorm events because they had lower engineering standards. In addition, kitchen houses, which are common in rural residential areas, significantly reduced the stability of the houses due to a height difference. Lam et al. [36] analyzed the windstorm resilience of rural houses in the Temerloh district, Peninsular Malaysia. The study showed that high wind speed events caused severe damage to roof sheathings and non-structural components. Aquino et al. [37] surveyed houses in two villages severely affected by the cyclone in Fiji, demonstrating building vulnerabilities caused by defects during the design and construction. Many physical measures to enhance building resilience to mitigate housing damage caused by hazards have been proposed [38,39]. Standohar-Alfano and van de Lindt [40] performed a probabilistic tornado hazard analysis of residential wood-frame roofs. The results showed that stricter residential building codes were crucial and beneficial in regions with a high tornado risk. Ripberger et al. [41] also proposed enhancing the building codes as a simple, inexpensive, yet highly efficient solution to reduce tornado-induced costs. Adelekan [42] assessed 69 houses damaged by a windstorm event in Ibadan by interviewing residents to evaluate the houses’ vulnerability. A survey of the damage and structural failures after the tornado revealed that maintaining the integrity of the façade and roof systems could change the external conditions of critical structural components [43]. Residential buildings face multiple hazards; thus, the same measure may have various effects on risk mitigation. For example, English et al. [44] pointed out that the elevation of houses could increase their resilience to flooding and their vulnerability to wind, indicating that the higher a residential building, the more damage it might incur. However, mitigating the damage may significantly reduce the effects of hazards rather than improving the buildings’ resistance to violent tornados. Reducing unnecessary damage to structures in lower-intensity tornados such as EF0-EF3 tornados can also result in fewer losses [45].

In summary, recent studies on housing damage during wind disasters have focused on building vulnerability to typhoons, hurricanes, tornados, and other events (e.g., the assessment of housing and environment damage, the structural performance of different building types, the influencing factors of losses, and the mitigation of the impact of wind disasters). Studies evaluating the influencing factors on housing damage have concentrated on the structural details of houses and wind speed. However, few studies have focused on examining environmental variables in disaster-hit locations, particularly in rural areas. The performance of different building types and structures in rural areas in China differs. Therefore, more research is needed. This study addresses the problem of disaster relief in rural areas, aiming to investigate the factors influencing housing damage during the Funing EF4 (Table A1) tornado on 23 June 2016, considering the building condition, tornado intensity, and environmental factors. A field investigation was conducted to determine the damage index of the affected villages and the tornado damage pattern. The relationship between the building damage and the building conditions, tornado intensity, and environmental factors was analyzed using a multinomial logistic regression model and Pearson’s correlation analysis. The remainder of this paper is organized as follows. Section 2 describes the study area and tornado and presents the field survey and data processing method. Section 3 describes the results including the characteristics of the damaged houses, visualization of the damage index, and the tornado damage pattern. The factors influencing housing damage are described. Section 4 provides the discussion and limitations of this study, and the conclusions and directions for future work are presented in Section 5. The results provide information to reduce losses in wind disasters and support disaster relief and prevention planning in rural areas.

## 2. Materials and Methods

### 2.1. Study Area and Tornado

**Figure 1:**
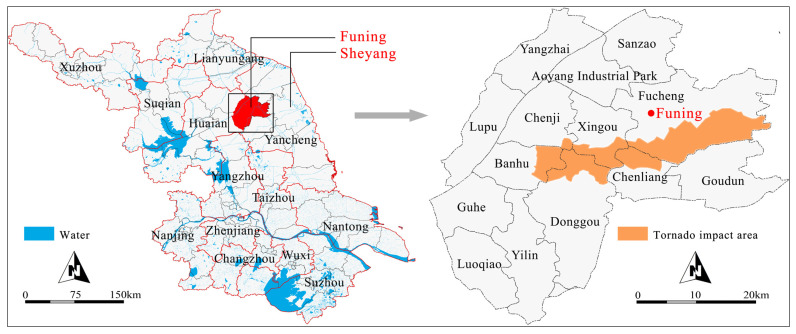
Location of Funing in Jiangsu Province (**left**) and tornado-affected areas in Funing (**right**).

Jiangsu Province is one of the two provinces most vulnerable to tornadoes [7]. Funing County is located in Yancheng City, Jiangsu Province, China (Figure 1). It has an area of 1439 km^2^ and a population of 1,120,000. There are 13 towns and four sub-district offices including the Funing Economic Development Zone, the High-Tech Zone, Jinsha Lake Tourism Resort, and Modern Service Park (http://www.funing.gov.cn/index.html accessed on 19 January 2022). Jiangsu and the neighboring Anhui are the two provinces with the most serious tornado disasters in China due to their geographical conditions. Jiangsu Province is located in the Jianghuai Plain. The hot and humid summers provide favorable conditions for severe convective weather and tornados. Additionally, a large proportion of the population and economy is exposed to high tornado risk, resulting in numerous missing persons and deaths and serious economic losses in Jiangsu Province in the past 30 years [46]. Funing is located in the center of Jianghuai Plain and Jiangsu Province; its geographical location and topographical features explain the occurrence of severe tornados. The 2016 tornado, which crossed the counties of Funing and Sheyang, was selected as a case study to improve the resilience of houses and reduce tornado-related risk.

**Figure 2:**
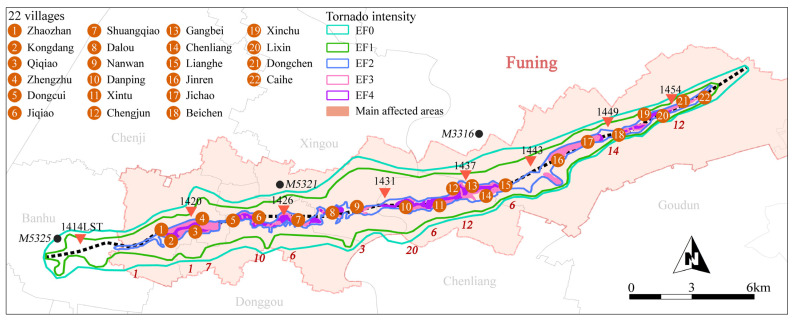
Tornado intensity and location of the 22 affected villages.

On 23 June 2016, 22 villages in Funing and seven villages in Sheyang were hit by extreme weather including a tornado, hail, lightning, and heavy rainstorm. The tornado event in Funing lasted from 14:00 to 15:00. The impacted area in Yancheng City was around 52 km long, and the tornado had an average width of 2.5 km, covering 269 km^2^. The total loss was approximately RMB 4377.39 million, and more than 45,509 people were affected by this disaster. Meng et al. [47] stated that the 2016 Funing tornado was the worst in the past 40 years in China. The tornado centerline is the dotted black line in Figure 2, the red area denotes the main affected villages, and the red triangles denote the locations of the 0.5° tornadic vortex signature (TVS) at different times (LST). The red numbers close to the southern edge of the damage swath represent the number of fatalities around the tornado center to the north of the number, and the black dots represent the in situ surface weather stations closest to the tornado damage swath during the disaster. The event was categorized as an EF4 tornado. The wind power exceeded the 17th grade, resulting in extreme weather, killing 99 people (98 in Funing), injuring at least 846 people, and destroying more than 2000 houses.

### 2.2. Data and Methods

#### 2.2.1. Field Survey of Housing Damage

The data were obtained during an emergency field survey of housing damage conducted by more than 180 experts in housing damage and emergency management. The experts were mainly composed of three parts: (1) 3 experts from the Jiangsu Provincial Department of Housing and Urban Rural Development; (2) 12 provincial expert groups from Jiangsu Suke Construction Technology Development Co, Ltd. and Jiangsu Jianyan Construction Engineering Quality and Safety Appraisal Co., Ltd., led by Weigen Yu, a national post disaster housing emergency assessment expert; and (3) numerous engineering and technical experts from Yancheng Architectural Design and Research Institute, Yancheng Construction Engineering Quality Inspection Center and other units. The survey was organized by the Jiangsu provincial government. Most of the authors of this study participated in the field survey and post-disaster recovery and reconstruction. Thus, a large amount of related information was obtained. The main purpose of the survey was to assess the extent of housing damage caused by the tornado; thus, the attention focused on the severely damaged areas. The experts were divided into 12 groups that participated in this investigation, and each group was composed of experts in five fields, including quality supervision, design, testing, supervision and identification. The survey process was as follows: (1) One day after the tornado, the investigation team established the unified framework for house damage assessment; (2) the groups visited each village to assess the state of housing damage based on a unified assessment standard, format, and technical requirements; (3) the conditions of the tornado-damaged houses such as the function, building area, building structure, and the number of stories were recorded and the damage degree was determined for each house to assess whether the house was suitable for repair. The damage categories were as follows. Slight damage (SD): the main part of the house was not damaged, and the living conditions were not affected; Dangerous and requiring immediate repair (DR): the houses were structurally damaged and were no longer habitable unless they are repaired; Unable to be repaired (UR): the house was structurally damaged, and the residents had to be relocated for construction.

The experts divided into 12 groups completed the investigation in seven towns including 22 villages in Funing County, and two towns including seven villages in Sheyang County. The investigation lasted three days. The total survey area was 864,300 m^2^ including 610,200 m^2^ of residential land (5907 households), 251,900 m^2^ of industrial land, and 2270 m^2^ of land for schools. It should be noted that residential buildings in the rural areas of Funing generally consist of two components: the living area and the kitchen and toilet. The living area usually has more stories and a larger area, whereas the kitchen and toilet are small areas with one story. The survey focused on the housing damage of both components.

#### 2.2.2. Variables and Data Processing

The analysis of the influencing factors on housing damage in wind disasters in existing studies provides a basis for selecting indicators in this study, including the wind intensity and path, building structure, number of stories, and the spatial distribution of buildings. The purpose of this study was to explore the influencing factors of housing damage in tornados. The housing damage variables (SD, DR, and UR) were the dependent variables. Recent research often considered the building conditions and the wind speed. Thus, in this study, we included these as the independent variables as well as the environmental factors of the village. Therefore, the independent variables included the building conditions, tornado intensity, and village environmental factors. The variables were as follows: (1) The building condition variables include the building area (BA), number of building stories (BSN1 and BSN2), and building material (masonry/concrete (BSMC), masonry (BSM), and other (BSO); (2) since the Funing tornado was an EF4 tornado, the tornado intensity had four categories (TS1, TS2, TS3, and TS4); (3) the village environmental factors included the village area (VA), floor area of village buildings (BAVB), and water area in the village (WAV). The variables are listed in Table 1.

The data on the housing conditions and damage assessment results were imported into a GIS database containing satellite images, topography, and detailed field information. The field survey provided 5381 data points of damaged houses from six towns (18 villages). The housing damage depends on the tornado intensity, housing conditions (structure, function, height, etc.), and environmental variables (VA, WAV). The data were derived as follows: (1) Data on housing damage were collected during the field survey; (2) building condition data were obtained from the field survey and GIS database; (3) data on the tornado intensity were obtained from the official meteorological agency; and (4) data related to the environmental factors (village environmental factors) were extracted from the GIS database.

Based on the 5381 data points, we established a damage index for a preliminary assessment of the housing damage from a macro-perspective (Equation (1)). The purpose was to obtain the distribution of damaged houses in the villages in the tornado path for different intensities and land use/land cover information. The results provided a damage pattern that clarified the relationship between housing damage and village pattern and provided a basis for establishing the analysis models based on specific site survey data. We calculated the area of residential land, the number of affected houses, and the number of houses in the villages for the intensity levels. Weights were assigned to the affected villages according to the number of buildings. The results were normalized to enable the comparison of the damage index in the villages.

(1) $$Damage\;Index=k\times \frac{BA}{TA}\times \frac{DH}{TDH}\times \frac{DL}{TDL}$$

where *k* is a weighting factor based on the percentage of houses in each village; *BA* is the building area; *TA* is the total area; *DH* is the number of damaged houses in each village; TDH is the total number of damaged houses; *DL* is the damage level in each village; *TDL* is the total damage level.

IBM SPSS Statistics 23.0 was used to establish the databases and perform statistical analyses. The PivotTable function was used to analyze the data on the 5381 houses obtained from the survey. The CrossTab function was then employed to determine the correlations between the severity of the damaged houses and other factors. Moreover, we converted the dependent variable to a categorical variable and conducted multinomial logistic regression analysis in SPSS to avoid subjectivity by the investigators during the data collection. The factors with low correlations with the dependent variable in the CrossTab results and statistically non-significant variables were removed. A total of 2557 data points were used in the multinomial logistic regression model (Equations (2) and (3)) to explain the relationship between housing damage and building conditions and tornado intensity. Since we had limited spatial data and different disaster conditions in the villages, the statistics from 12 villages were used in canonical correlation analysis to determine the relationship between housing damage and village environmental factors. Data statistics are shown in Table 2.

(2) $$Logit\left(pi\right)=\frac{1}{1+exp\left(-pi\right)}$$

(3) $$In\left(\frac{pi}{1-pi}\right)=Beta\_0+Beta\_1\times X\_1+\ldots +B\_k\times K\_k$$

where *logit*(*pi*) is the dependent variable, and *X* is the independent variable. The beta parameter is commonly estimated via the maximum likelihood estimation.

## 3. Results

### 3.1. General Characteristics of Damaged Houses

The statistics of 5381 tornado-damaged houses were collected from six towns including 18 villages, but predominantly from Banhu, Chenliang, Shuoji, and Xingou. More than 98% of houses were residential buildings (rather than commercial buildings), pigsties, industrial buildings, and public service facilities. In addition, more than 99% of the houses were owned by the occupiers. Nearly half of the houses had an area of 50 m^2^ to 100 m^2^, and 32.69% had areas of 100 m^2^ to 200 m^2^. A total of 3362 damaged houses were masonry-concrete structures or other hybrid structures, and 1408 were masonry structures. More than 4000 houses had one story, and 1266 houses had two stories; some were totally destroyed and could not be measured. After the field investigation, 2227 houses were classified as SD, 884 houses as DR, and 1844 houses as UR.

**Figure 3:**
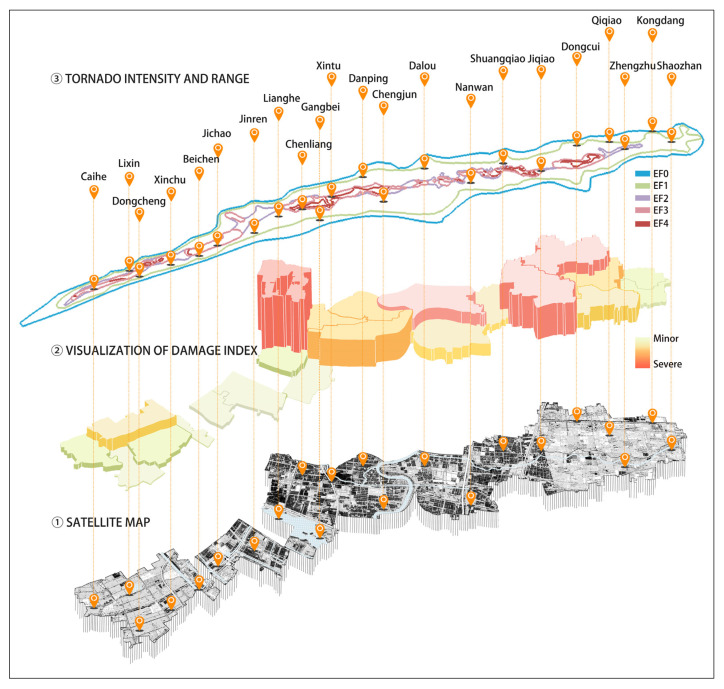
The damage index, tornado intensity, and range.

It is commonly assumed that houses are more severely damaged in areas with high tornado intensity. However, the results (Figure 3) showed that the extent of damage to the villages did not correspond entirely to the tornado intensity of the location (i.e., areas with high tornado intensity did not necessarily have more severely damaged houses). It is also commonly believed that in rural areas where the quality of housing is generally not as good as in cities, areas with a high building density may have more severe damage to houses during a tornado [36]. However, the observed damage pattern showed that the building density was not related to the severity of damage to houses (i.e., villages with a high building density were not necessarily more severely damaged during the tornado). Based on the results of the preliminary analysis, we analyzed the damage to the building units and the environment using information from the site survey.

The results of the regression analysis of the 2557 data points on housing damage (Table 3) showed that 1025 houses suffered SD (40.1%) and 1007 were UR (39.4%). It was found that 1980 of the damaged houses had one story (77.4%), 67.6% were masonry-concrete structures, and 21.7% were masonry structures. Different tornado intensities caused different damage levels to houses. The EF3 tornado damaged the most houses (1211, 47.4%), followed by the EF1 (30.9%), EF4 (17.9%), and EF2 tornados.

### 3.2. Factors Related to Houses and Tornado Intensity

The overall fit of the multinomial logistic regression model was initially analyzed using fitness indicators most frequently reported in previous studies [48] including chi-square (χ^2^), degrees of freedom (df), χ^2^/df, and significance, with values of 2842.742, 2460, 1.156, and 0.000, respectively. The recommended χ^2^/df was between 1 and 3; therefore it was within the acceptable range (not exceeding 5). Therefore, the regression model provided a good fit for the survey data. Three key findings were obtained from the multinomial logistic regression analysis of the 2557 data points on housing damage (Table 4). (1) Houses with two stories were more likely than houses with 1-story to have SD or be DR, indicating that 2-story buildings in rural areas were more likely to suffer severe damage. (2) Masonry houses were more likely to have SD than masonry-concrete houses and other hybrid structures. In addition, the other hybrid houses and masonry-concrete houses were more likely to be DR than masonry houses, indicating that masonry houses in rural areas tend to suffer less damage than hybrid houses. (3) The BA was positively correlated with houses in the SD and DR categories, revealing that the larger the BA, the more severe the damage.

The results demonstrate a weak correlation between the level of housing damage and tornado intensity. We mainly focused on the SD and DR categories. The multinomial logistic regression analysis showed that houses classified as SD were more likely to have been hit by the EF4 tornado than by the EF3 tornado. However, houses classified as SD or DR were more likely to have been damaged by the EF1 or EF2 tornados than by the EF4 tornado. Thus, the EF3 tornado caused less damage to houses than the EF4 tornado in the rural areas of Funing, although the damages were more likely to be linked to the EF1 or EF2 tornado than the EF4 tornado. The probable reason is the location where the tornado struck. In addition, there are different housing distributions and densities in rural areas, indicating that areas hit by the EF1 or EF2 tornados may have been damaged more seriously than those hit by the EF4 tornado (Table 4).

### 3.3. Factors Related to the External Environment

**Figure 4:**
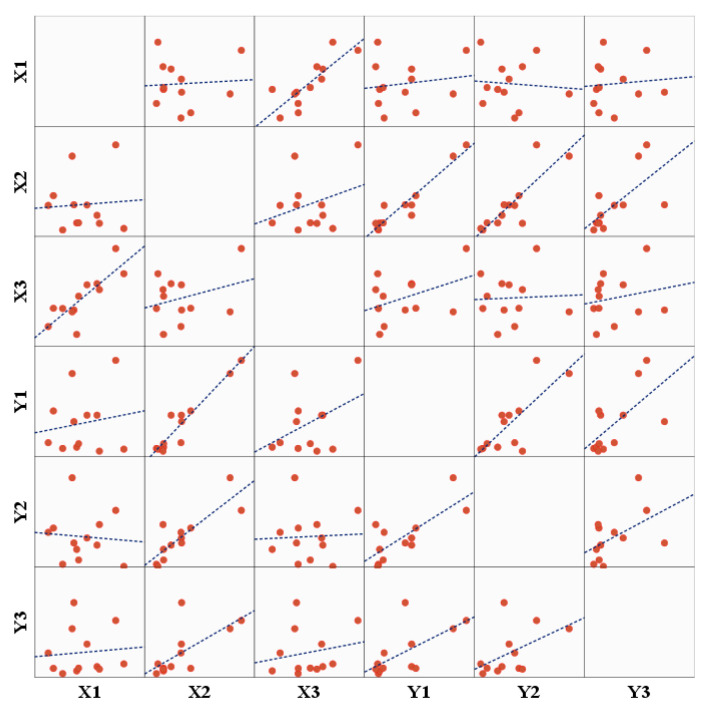
The scatterplot matrix of the village environmental factors and housing damage.

In order to analyze the relationship between the external environment of the village (village environmental factors) and housing damage, considering the differences in housing damage between the villages and the limited spatial data, the housing damage statistics from 12 villages (Table 2) were used for further analysis. According to the types of data, the factors were divided into two groups (Set 1 and Set 2). Set 1 included VA (*X*_1_), BAVB (*X*_2_), and WAV (*X*_3_); Set 2 included SD (*Y*_1_), DR (*Y*_2_), and UR (*Y*_3_). A scatterplot matrix was derived based on the data, and canonical correlation analysis was performed. This method determines the correlations between numerous variables. The canonical correlation coefficient measures the strength of two canonical variates. The scatterplot matrix represents the general characteristics of the relationship between the variables in the two sets (Figure 4). The canonical correlation model revealed a significant correlation between the two sets of data, with correlation and significance values of 0.975 and 0.003, respectively, indicating that Set 1 was positively correlated with Set 2 (Table 5). The sets can be expressed as:

Set1 = −0.035 × *X*_1_ − 0.999 × *X*_2_ + 0.004 × *X*_3_, (4)

Set2 = –0.706 × *Y*_1_ − 0.275 × *Y*_2_ − 0.099 × *Y*_3_. (5)

The relationship between the level of damage to the houses and the village external environmental factors can be interpreted from Equations (1) and (2). According to the statistical significance level, the proportion of houses with SD was positively correlated with the water area density of the village, whereas this was not the case for the other two categories (DR and UR). The larger the WAV, the less housing damage it suffered. The VA area was associated with housing damage in the SD category, indicating that the size of a building increased with the VA, increasing the housing damage. In addition, the larger the VA, the higher the level of damage to the houses. Houses with SD exhibited the highest correlation with village environmental factors, whereas houses in the UR category showed a weak correlation. Thus, the serious damage to houses from this tornado might have been related to factors other than environmental factors in the rural areas of Funing.

## 4. Discussion and Limitations

The mining of influencing factors of losses resulting from disasters is of great significance in reducing losses, improving resilience, and building a sound regional disaster prevention capability in future emergencies. In order to analyze the influencing factors of housing damage in relevant villages in the Funing tornado, three types of independent variables were considered including building conditions, tornado intensity, and village environmental factors to analyze the factors influencing housing damage in different villages caused by the Funing tornado. A damage index was established, and the damage pattern was examined to obtain a preliminary assessment of the tornado damage to houses. Subsequently, multinomial logistic regression analysis was used to determine the relationships between the degree of housing damage (SD, DR, and UR) and the independent variables. From the perspective of disaster relief, the findings of this study have practical significance for disaster relief and provide insights into reducing housing losses and casualties during wind disasters in rural areas.

The building area, building material, and the number of building stories were correlated with the housing damage level [12]. Generally, the larger or taller a house, the more severe the damage. The post-disaster field investigation showed that some 2-story buildings were only slightly damaged, but most of these buildings were constructed in the past 10 years. Thus, their strong structural performance made them less vulnerable to tornados. In other words, the age of the house may also affect housing damage. This factor was not considered in this study. In addition, the building areas of rural houses in China are similar because the area is typically based on a standard, and expanding the BA is not permitted. The difference between the building areas is related to the ancillary facilities. A house with more ancillary facilities has a larger BA. Therefore, the impact of the BA on housing damage should be further explored. As previously mentioned, the construction age also affects housing damage. In general, the older the building, the lower its structural performance is in a disaster, which is true for tornados. Moreover, the building structure details (roofs, entrances, etc.), which were not covered here, were associated with housing damage in previous studies [18,29].

In the view of the impact of tornado intensity on house damage, the damage caused by high-intensity tornados is typically considered higher than that of low-intensity tornados for houses with the same area. In the current study, houses were more likely to be slightly damaged by EF4 tornados than by EF3 tornados, or by EF1 or EF2 tornados than by EF4 tornadoes. There are two explanations for this. First, there are differences in the building distribution between villages. If a building is very close to another, the wind speed may increase in strong wind conditions, causing the buildings to be damaged [15]. Low-intensity tornados passing through areas with dense buildings cause more damage to houses than high-intensity tornados passing through areas with sparse buildings. In addition, the construction quality must also be considered. If the building quality of a village is low, low-intensity tornados can cause substantial damage, while the losses caused by high-intensity tornados may be the same. A study by Nateghia [49] indicated that an F2 tornado was strong enough to destroy a wood-frame or nonreinforced masonry building. Most of the damaged houses were in the rural areas of Funing, and the low-quality houses could not withstand the tornado. Therefore, the correlation between the housing damage and the tornado intensity was weak for tornados stronger than EF2 because most houses in Funing were already severely damaged by the EF2 tornado.

The degree of damage to houses is, to some extent, related to the village environmental factors. In rural areas, trees are generally planted near the water, potentially protecting the houses. Moreover, the WAV in the village may have influenced the distribution and spatial pattern of the buildings, affecting the extent of tornado damage to the buildings. In addition, a large waterbody can affect the tornado strength and housing damage [50]. Villages with a large VA typically have more buildings and a larger BAVB. The regression analysis showed that the number of houses with SD was associated with the total BA, which may be related to the path of the tornado (i.e., a low-intensity tornado passing through areas with high building densities). A village has a complex environment, and numerous factors should be considered such as topography, landform, residential settlement form, and green coverage. Since these factors may influence the housing damage in a tornado, they need to be explored in future studies.

Recent studies and our case study on the Funing tornado indicate many complex variables affecting housing damage in tornados, particularly in rural areas, where there are no fixed building codes, low structural performance of buildings, limited experience in constructing buildings resistant to wind disasters, and other risk factors. The current study analyzed the influencing factors of housing damage in a tornado using a multinomial logistic regression model. The results provide new information on reducing house losses in wind disasters in rural areas. However, knowledge of the relationship between housing damage and various dimensional factors remains limited, and this study has some limitations that may affect the results. In addition to the areas of improvement mentioned above, the classification of the houses by the investigators may have been subjective and may not reflect the actual level of destruction due to the limited investigation time and post-disaster reconstruction. Moreover, the data for this study were obtained from the post-disaster emergency survey, which had the primary goal of emergency response and the rapid resettlement of affected residents after the disaster. Therefore, there may be some limitations in the comprehensiveness of the data collection. In a wind disaster, housing damage is a result of a combination of man-made and natural factors. Therefore, to analyze the variables that affect the losses, it is necessary to consider the building conditions, disaster intensity, and external environmental factors as fully as possible. In any case, from the perspective of disaster relief practice in rural areas, this study is a reference in reducing losses when a potential disaster occurs.

## 5. Conclusions

Rural areas are vulnerable to disasters and tend to suffer more severe losses in destructive disasters than urban areas. An EF4 tornado occurred in Funing on 23 June 2016, killing 99 people, injuring at least 846 people, and destroying more than 2000 houses. We conducted a field investigation and analyzed the influences of the building condition, tornado intensity, and village environmental factors on housing damage using a multinomial logistic regression model and Pearson’s correlation analysis. The main findings of this study were as follows. (1) 2-story houses and masonry houses were more likely to have SD or be DR. The BA was positively correlated with houses in the SD and DR categories, indicating that the larger or taller a house, the more severe the damage. (2) Houses classified as SD were more likely to have been hit by the EF4 tornado than by the EF3 tornado, and the houses were damaged more by the EF1 or EF2 tornados than the EF4 tornado. This finding indicated that the level of housing damage was not strongly correlated with the tornado intensity in the Funing case, which may be related to the building density in the path of the tornados with different intensities. (3) Houses classified as SD had the highest correlation with environmental factors in the village. The number of damaged houses increased with the VA and BA. The proportion of houses with SD was positively correlated to the WAV, unlike the DR and UR houses. Moreover, the larger the WAV, the lower the housing damage.

The variables affecting housing damage in tornados are complex. This study took the rural areas in the Funing tornado as the study object to conduct a multinomial logistic regression analysis on the influencing factors of housing damage. Based on the results, the practical implications for improving the housing resilience of rural areas in wind disasters are as follows. First of all, building structures in wide rural areas should be enhanced, particularly for houses with individual kitchen and toilet areas; second, the spatial distribution of residential buildings in counties should follow a scientific basis for rural planning, as environmental factors are also associated with the housing damage in wind disasters; furthermore, as there are differences in building conditions, structure types, and disaster environments in rural areas across regions and ethnic groups, more research should be carried out to determine additional potential influencing factors on housing damage, thereby increasing the disaster resilience of rural areas.

## Figures and Tables

**Table 1:** Analysis variables.

Variable Type	Variable Dimension	Variables
Independent variable	Building conditions	(BA). Building area (m^2^)
		(BSN1). Building story number = 1
		(BSN2). Building story number = 2
		(BSMC). Building structure = masonry-concrete
		(BSM). Building structure = masonry
		(BSO). Building structure = other hybrid
	Tornado intensity	(TS1). Estimated tornado intensity = EF1
		(TS2). Estimated tornado intensity = EF2
		(TS3). Estimated tornado intensity = EF3
		(TS4). Estimated tornado intensity = EF4
	Village environmental factors	(VA). Village area (m^2^)
		(BAVB). Building area of village buildings (m^2^)
		(WAV). Water area in the village (m^2^)
Dependent variable	Housing damage	(SD). Slight damage
		(DR). Dangerous and requiring immediate repair
		(UR). Unable to be repaired

**Table 2:** Variable data of the selected 12 villages.

Village	VA	BAVB	WAV	N. of SD	N. of DR	N. of UR
Caihe	3,369,866	5169	461,359	34	4	15
Chengjun	5,452,885	17,001	760,903	168	31	43
Dalou	6,583,346	73,776	1,186,041	389	80	229
Danping	4,844,602	25,298	747,055	168	41	135
Dongcui	7,076,906	6355	881,643	31	1	54
Gangbei	4,219,727	10,850	147,756	39	25	26
Kongdang	2,493,275	24,981	243,022	57	49	98
Lixin	4,335,627	10,913	609,406	53	10	37
Lianghe	2,807,860	32,862	463,656	185	55	36
Nanwan	5,596,270	10,491	689,044	23	60	33
Shuangqiao	4,051,253	25,475	442,333	142	34	301
Xintu	3,946,710	64,705	420,572	336	126	196

**Table 3:** Descriptive statistics of housing damage.

Characteristic		Frequency	Percentage
Damage classification	SD	1025	40.1%
	DR	525	20.5%
	UR	1007	39.4%
Number of stories	1	1980	77.4%
	2	577	22.6%
Building material	Masonry-concrete	1728	67.6%
	Masonry	556	21.7%
	other	273	10.7%
Estimated tornado intensity	EF1	789	30.9%
	EF2	99	3.9%
	EF3	1211	47.4%
	EF4	458	17.9%
Valid		2557	100.0%
Missing		0	
Total		2557	

**Table 4:** Parameter estimates obtained from the multinomial logistic regression model.

Dependent Variable	Independent Variable	*B*	*Sig*.	*Exp*(*B*)
Slight damage (SD)	Intercept	0.895	0.002	
	BA	0.003	0.025	1.003
	BSN1	−1.751	0.000	0.174
	BSN2	0	.	.
	BSMC	0.151	0.412	1.163
	BSM	0.903	0.000	2.466
	BSO	0	.	.
	TS1	0.379	0.007	1.460
	TS2	2.298	0.000	9.950
	TS3	−0.487	0.000	0.614
	TS4	0	.	.
Dangerous and requiring immediate repair (DR)	Intercept	0.465	0.148	
	BA	0.005	0.002	1.005
	BSN1	−1.024	0.000	0.359
	BSN2	0	.	.
	BSMC	−1.277	0.000	0.279
	BSM	−0.416	0.032	0.660
	BSO	0	.	.
	TS1	0.395	0.035	1.484
	TS2	2.066	0.000	7.894
	TS3	0.116	0.516	1.123
	TS4	0	.	.

*B* is the coefficient for the constant in the null model; *Exp*(*B*) is the exponentiation of the *B* coefficient, which is an odds ratio; *Sig.* is the *p*-value. The null hypothesis is rejected if *p* < 0.05.

**Table 5:** Standardized canonical correlation coefficients.

Set	Variable	1	2	3
Set 1	*X*_1_	–0.035	–0.759	–1.707
	*X*_2_	–0.999	–0.470	–0.195
	*X*_3_	0.004	1.633	1.085
Set 2	*Y*_1_	–0.706	1.581	0.431
	*Y*_2_	–0.275	–1.453	0.510
	*Y*_3_	–0.099	–0.420	–1.244

## Data Availability

Not applicable.

## Appendix A

**Table A1:** EF rating by 3-s Gust (mph).

EF Rating	3-s Gust (mph)
0	65–85
1	86–110
2	111–135
3	136–165
4	166–200
5	Over 200

EF SCALE WINDS: The EF scale is a tornado rating based on the estimated wind speeds (not measurements) and associated damage. It uses three-second gusts estimated at the point of damage based on a judgment of eight levels of damage to the 28 indicators. These estimates vary with height and exposure. Important: The 3-s gust is not the same wind speed as in standard surface observations. Standard measurements are taken by weather stations in open exposures, using a directly measured “one-minute mile” speed (https://www.weather.gov/oun/efscale accessed on 27 May 2022).
